# Malaria and environmental, socioeconomics and public health conditions in the municipality of São Félix do Xingu, Pará, Eastern Amazon, Brazil: An ecological and cross-sectional study

**DOI:** 10.1590/0037-8682-0502-2022

**Published:** 2023-04-14

**Authors:** Nelson Veiga Gonçalves, Bruna Costa de Souza, Marília de Souza Araújo, Emerson Cordeiro Morais, Bruma Gouveia de Melo, Silvana Rossy de Brito, Maria de Fátima Pinheiro Carrera, Simone Beverly Nascimento da Costa, Taiana Moita Koury Alves, Thalita da Rocha Bastos, João Simão de Melo, Claudia do Socorro Carvalho Miranda

**Affiliations:** 1 Universidade do Estado do Pará, Laboratório de Epidemiologia e Geoprocessamento da Amazônia, Belém, PA, Brasil.; 2 Universidade Federal Rural da Amazônia, Instituto Ciberespacial, Belém, PA, Brasil.; 3 Universidade Federal do Pará, Instituto de Ciências da Saúde, Belém, PA, Brasil.; 4 Centro Universitário da Amazônia, Belém, PA, Brasil.

**Keywords:** Malaria, Spatial analysis, Epidemiology, Deforestation

## Abstract

**Background:**

Malaria is a parasitosis conditioned by several factors. This study sought to analyze the spatial distribution of malaria considering environmental, socioeconomic, and political variables in São Félix do Xingu, Pará, Brazil, from 2014 to 2020.

**Methods:**

Epidemiological, cartographic, and environmental data were obtained from the Ministry of Health, Brazilian Geographical and Statistical Institute, and National Space Research Institute. Statistical and spatial distribution analyses were performed using chi-squared tests of expected equal proportions and the kernel and bivariate global Moran’s techniques with Bioestat 5.0 and ArcGIS 10.5.1.

**Results:**

The highest percentage of cases occurred in adult males with brown skin color, mainly placer miners, with a primary education level, living in rural areas, who were infected with *Plasmodium vivax* and with parasitemia of two or three crosses as diagnosed by the thick drop/smear test. The disease had a non-homogeneous distribution, with distinct annual parasite indices associated with administrative districts and clusters of cases in locations with deforestation, mining, and pastures close to Conservation Units and Indigenous Lands. Thus, a direct relationship between areas with cases and environmental degradation associated with land use was demonstrated, along with the precarious availability of health services. Pressure on protected areas and epidemiological silence in Indigenous Lands were also noted.

**Conclusions:**

Environmental and socioeconomic circuits were identified for development of diseases associated with precarious health services in the municipality. These findings highlight the need to intensify malaria surveillance and contribute to the systematic knowledge of malaria’s epidemiology by considering the complexity of its conditioning factors.

## INTRODUCTION

Malaria is an infectious parasitosis caused by protozoans of the genus *Plasmodium,* transmitted to humans by female mosquitoes of the genus *Anopheles*. This disease is epidemiologically important because of its high morbidity and mortality in endemic areas and often evolves into severe forms when not treated in a timely fashion. It is considered a major public health issue in the Amazon region of Brazil, accounting for 89.3% of cases of the disease reported in Brazil in 2019. This high percentage may also be under-reported owing to limitations in diagnosing and recording cases^1^ ^-^ ^4^.

Over the last decade, Brazil averaged approximately 145 thousand cases of malaria per year, mostly in the Amazon region, due to the environmental and socioeconomic characteristics found in this region^3^. During this period, the state of Pará reported a high number of cases, and the southeastern region of the state showed a significant increase in reported cases. This may be associated with environmental degradation processes related to land use and cover, especially near Conservation Units (CU) and Indigenous Lands (IL). These areas have historically enabled the preservation of the natural environment and health of traditional people because of their protected status^5^ ^-^ ^7^.

The municipality of São Félix do Xingu recorded 407 cases of malaria and high rates of deforestation from 2014 to 2020. The deforestation rates led to São Félix do Xingu's inclusion in the list of municipalities with the highest levels of vegetation suppression in Brazil, with an increase of approximately 1.264 km² in deforestation. Although Protected Areas (PA) covered approximately 72% (62.000 km²) of its territory were created in 2006 and encompass CU at 19.33% (16.274 km²) and IL at 53.34% (44.902 km²), these areas are under constant human pressure to exploit their natural resources. The PA still contained forests with low levels of degradation, albeit under pressure, mainly along their edges^8^ ^-^ ^10^.

This socioenvironmental scenario establishes various potential circuits for malaria transmission in the municipality, whose administrative districts present different annual parasite indices (API) related to the precarious nature of healthcare services. This represents a major challenge for public health, in terms of effective disease monitoring and control. Therefore, epidemiological and environmental surveillance of malaria will require analyses of its occurrence related to biotic, abiotic, socioeconomic, and political variables to establish a systematic epidemiology defining the relationship between causal and spatial dependence of the occurrence of malaria and its conditioning factors.

Spatial data analysis has been widely used, especially on the local scale, for studies of several infectious diseases because it enables the identification of possible relations of dependence between spatialized variables^11^ ^-^ ^13^. With that in mind, this study sought to contribute towards establishing a systematic memory of the spatial distribution of malaria based on an analysis of its occurrence related to socioeconomic, environmental, and public health variables in the municipality of São Félix do Xingu, Pará state, from 2014 to 2020.

## METHODOLOGY

This cross-sectional and ecological study of confirmed malaria cases employed epidemiological data (sex, age range, ethnicity, occupation, educational level, residence area, diagnostic exam, *Plasmodium* species, and parasitemia) obtained from the Disease Notification System. It also made use of public health data from Coverage by Health Establishments (CHE), the National Registry of Health Establishments, Basic Health Care Coverage (BHCC), and the Basic Care Information and Management System, all of which were from the Ministry of Health. Environmental data (land use and cover) were obtained from the TerraClass Project of the National Institute for Space Research. Cartographic data from IL (Apyterewa, Bacaja, Kayapó, Badjonkore, and Menkragnoti) and CU (Serra do Pardo National Park, Triunfo do Xingu Environmental Protection Area, and Tapirapé-Aquiri National Park) were obtained from the databases of the National Indian Foundation and the Brazilian Institute of Environment and Natural Resources. Population data and geographical locations of the administrative districts (Ladeira Vermelha, Lindoeste, Nereu, Sudoeste, Taboca, and Sede Municipal) and their localities (Pista da Liberdade, Lindoeste, Nereu, Campo Verde, Farinha, São Raimundo, Solar das Águas, and São Francisco) were collected from the Demographic Census databases (2020 estimate) at the Brazilian Institute of Geography and Statistics.

Locations of malaria cases, types of land use and cover (pasture, urban areas, mining, secondary vegetation, and forest), and IL and CU were georeferenced in the field using a global positioning system receiver. Inconsistencies and incomplete information were removed from the data, which were later indexed in a geographic database. Analysis of the API historical series was performed using the linear trend technique during the study period. In the descriptive analysis, percentage calculations and the non-parametric chi-squared test of expected equal proportions with a significance of <0.05% were performed^14^. All analyses were performed using the free Bioestat 5.0 software program for Biostatistical analysis (José Ayres and others and was funded by CNPq, Brazil) using the protocol recommended by the Ministry of Health to calculate API^15^.

The kernel technique was used to analyze the spatial distribution of the disease by identifying areas with the highest concentrations, and the bivariate global Moran's Index (I) was employed to describe the possible spatial dependence in areas with different types of land use and cover, considering a direct relationship for I>0 and an inverse relationship for I<0. Values close to the variation limits (-1 and 1) were considered strong^16^. These analyses were performed and expressed on a thematic map using the ArcGIS 10.5.1 software.

This study was approved by the Research Ethics Committee of the State University of Pará (3.950.486/2019) in line with the rules of Resolution no. 466/2012 of the National Health Council.

## RESULTS

**FIGURE 1:**
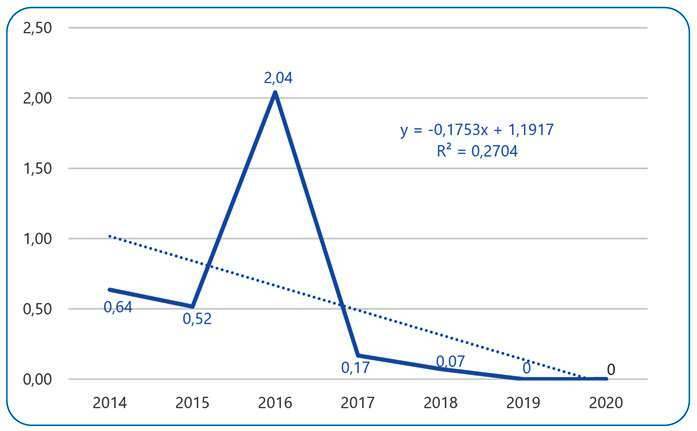
Annual Parasite Index of Malaria in São Félix do Xingu, Pará, Brazil, from 2014 to 2020. **Source:** Research Protocol, 2022.

Four hundred and seven confirmed cases of malaria in the São Félix do Xingu area were analyzed. A fluctuation in the API was observed throughout the study period (2014-2020), with an outbreak of the disease in 2016 and an overall downward linear trend until 2020, as shown in Figure 1. Non-homogeneous occurrence of the disease was also observed in the municipal administrative districts.

The epidemiological profile of the persons affected showed a higher percentage of males (80.10%), individuals in the 19- to 59-year-old age range (76.17%), those with brown skin color (n=169; 41.52%), those with up to an elementary school education (80.84%), those working as placer gold miners (65.85%), and those living in rural areas (76.17%). Clinically, almost all examinations were performed using the thick drop/smear method (91.65%), with parasitemia at two or three crosses (“+ +/+ + +”) (65.36%). The most common species was *Plasmodium vivax* (90.17%), as shown in Table 1.

**TABLE 1:** Epidemiological profile of confirmed cases of malaria in the municipality of São Félix do Xingu, Pará, Brazil, from 2014 to 2020.

Variables		n = 407	%	*P-value
Sex	Female	81	19.90	< 0.0001
	Male	326	80.10	
Age Group	Child/ Teenager (0 - 18 years)	90	22.11	< 0.0001
	Adult (19 - 59 years)	310	76.17	
	Elderly (≥60 years)	7	1.72	
Race/Ethnicity	White	102	25.06	< 0.0001
	Indigenous	67	16.46	
	Brown/Mixed race	169	41.52	
	Black	69	16.95	
Education	Elementary School	329	80.84	< 0.0001
	High School	24	5.90	
	Illiterate/Ignored	30	7.37	
	N/A	24	5.90	
Occupation	Agriculture	23	5.65	< 0.0001
	Mining	268	65.85	
	Livestock	14	3.44	
	Other	102	25.06	
Zone	Urban	97	23.83	< 0.0001
	Rural	310	76.17	
Diagnosis	Thick Drop/Smear	373	91.65	< 0.0001
	Quick test	34	8.35	
Parasitemia	Up to 1/2 +	63	15.48	< 0.0001
	+	51	12.53	
	++ / +++	266	65.36	
	Ignored	27	6.63	
*Plasmodium* species	*Plasmodium falciparum*	16	3.93	< 0.0001
	*Plasmodium vivax*	367	90.17	
	Unknown	24	5.90	

* p<0.05 (Chi-square, adherence). **n:** number of cases. **Source:** Research Protocol 2022.

**FIGURE 2:**
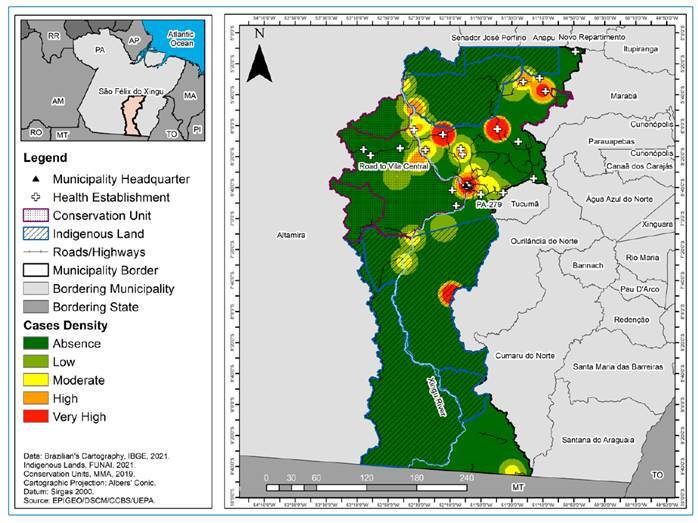
Spatial distribution of malaria and coverage by health establishments in the municipality of São Félix do Xingu, Pará, Brazil, from 2014 to 2020. **Source:** Research Protocol, 2022.

API analysis in the administrative districts of São Félix do Xingu showed that Taboca (18.98) and Ladeira Vermelha (14.89) had a medium risk for the disease. In Sudoeste (7.28) and Lindoeste (2.53), this index was low, and in Sede Municipal (0.91) and Nereu (0.54), it was very low. Regarding the spatial distribution of malaria, the kernel density estimation technique showed a non-homogeneous pattern for its occurrence in different localities in the municipality. In São Raimundo (204) and Pista da Liberdade (116), a very high density of cases was observed, especially close to IL and CU. This indicator was moderate in Lindoeste (24) and Sede Municipal (45), and low in Solar das Águas (5), São Francisco (3), Campo Verde (3), Nereu (4), and Farinha (3), as shown in Figure 2. 

The BHCC was found for only 45.65% of the inhabitants in the municipality. For CHE, 60.20% were found in urban areas, and only 39.80% were found in rural areas. With this in mind, approximately 76% of the malaria cases occurred in rural areas, while the remaining 24% were found in urban areas. This indicates a major inequity in healthcare associated with the distribution of the BHCC and CHE programs, with a greater supply in urban areas and precarious coverage in rural areas. This has implications for the epidemiological scenario of malaria, as demonstrated in Figure 2.

**FIGURE 3:**
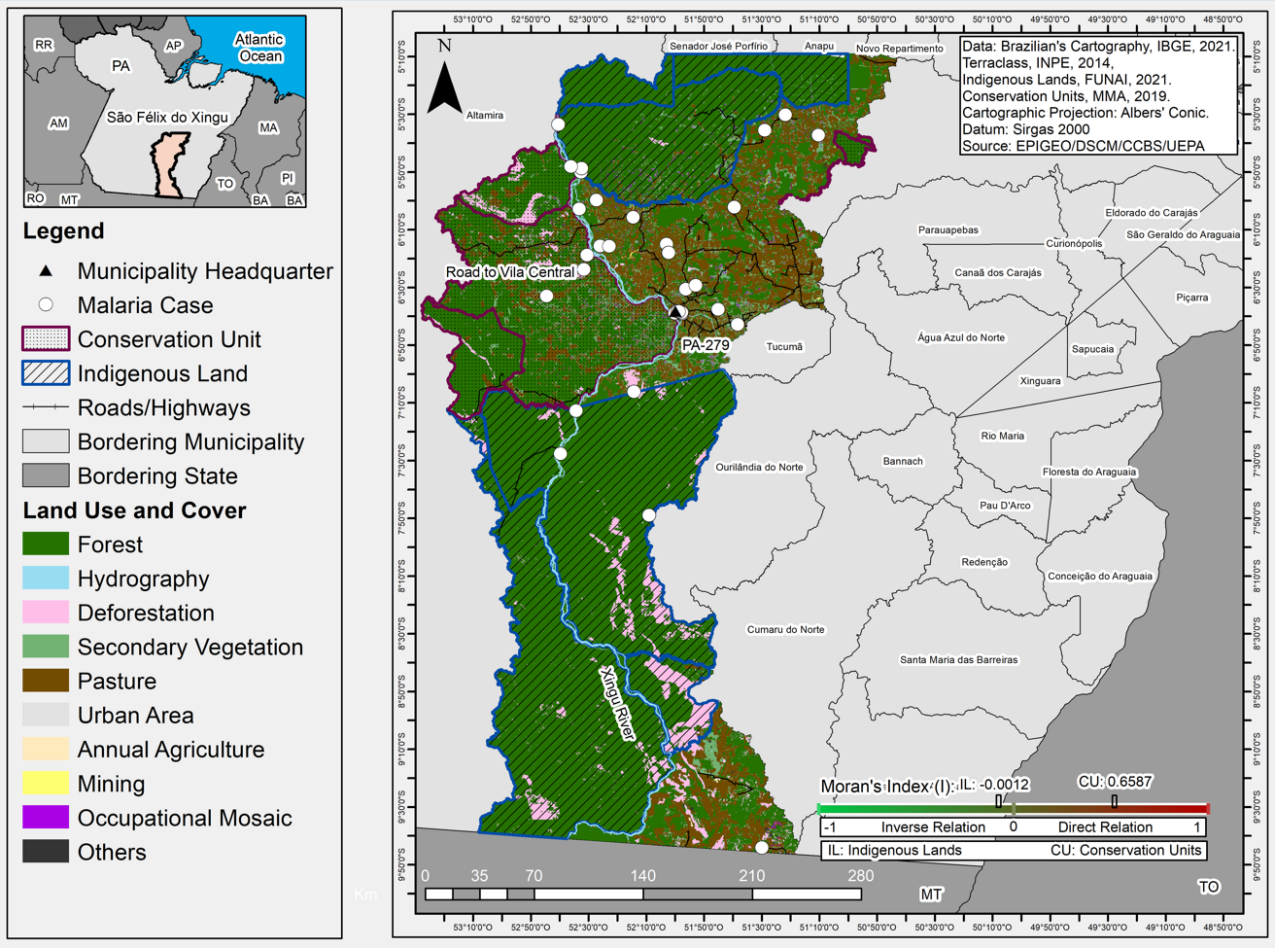
Malaria distribution and land use and cover in the municipality of São Félix do Xingu, Pará, Brazil, from 2014 to 2020. **Source:** Research Protocol, 2022.

Analyses of spatial dependency using the bivariate global Moran's Index for areas with cases of malaria and several types of land use and cover showed significant spatial autocorrelations, which were mainly found in the protected territorial units in the municipality. These correlations were direct and strong (0.6587) in CU and inverse and weak (-0.0012) in IL. These spatial relations presented evidence of association with the results of human activities, such as secondary vegetation, pastures, and placer mining, as shown in Figure 3.

## DISCUSSION

In general, analyses of the results showed that during the study period, malaria in São Félix do Xingu was in line with epidemiological scenarios for the disease in the Amazon, especially in relation to the significant reduction in cases that occurred in 2020, possibly as a result of the reduction in health surveillance actions observed during the COVID-19 pandemic^17^ ^,^ ^18^. The fact that the disease was significantly higher in male individuals at a productive age (19-59 years) may be related to work under unhealthy conditions performed by adult men. This work also involves the lack of use of individual protection (such as repellent and appropriate clothing), especially in illegal mining areas and ranching, which exposes these workers to factors associated with various infectious diseases, such as malaria^7^ ^,^ ^11^ ^,^ ^19^.

The higher occurrence of the disease among brown-skinned people in the municipality may be related to the historical formation of the Amazon, where approximately 73% of the population self-declared as belonging to that ethnic classification as a result of the intense process of intermingling between indigenous peoples with Europeans and Africans going back to the beginning of colonization^20^ ^-^ ^22^. The low level of schooling observed is also a factor associated with malaria occurrence, since it is related to poverty and the socioeconomically precarious condition of populations living in localities with a high density of cases^23^ ^,^ ^24^. This implies limitations in terms of access to and appropriate knowledge of the disease, which might otherwise be obtained in a school environment and health programs, that is, information on its forms of transmission, diagnosis, and treatment. It also influences the perception of individuals who are infected or exposed to the risk of infection due to environmental factors^11^ ^,^ ^19^.

Thus, rural populations, as well as the indigenous persons observed in the study area during fieldwork activities, who find themselves in social precariousness and lacking access to information about malaria, were found to have a major level of unawareness regarding means for preventing malaria. In this regard, incorporating health education into school curricula, especially for historically vulnerable populations, may become an important instrument for reducing their exposure to the risks of becoming ill from malaria.

Inequities in health coverage related to low BHCC and CHE availability in the municipality, which is higher in the urban environment and lower in rural areas, is a major factor contributing to the high density of malaria cases in São Raimundo, Pista da Liberdade, and on the borders of IL. This fact suggests that an epidemiological scenario with an active transmission circuit has been established for the disease, which is conditioned by the precarious availability of public services in areas where a conjunction of socioeconomic and environmental risk factors is observed. This indicates a need to expand health surveillance strategies to mitigate the impact of malaria in the municipality.

The epidemiological scenario of the disease is further aggravated by the precarious supply of epidemiological and environmental surveillance services, mainly in rural areas in the municipality and above all in the IL, which complicates the objective of reducing the number of malaria cases in those areas, given the difficulties in access and the remoteness of their locations. The establishment and continuation of this reduce service creates a scenario of social inequity related to a historical deficit in healthcare for these traditional populations, marked by their invisibility and reflected in the inefficient delivery of public health services to them, even though they have a constitutionally guaranteed right to adequate healthcare.

The fact that the pattern for malaria in São Félix do Xingu is rural and occupational and involves placer mining as well as agriculture and ranching activities suggesting an association of the disease with unhealthy working conditions due to the constant process of removing native vegetation to create pastures. This has been aggravated over the last few decades due to the intense migration of people to work in these activities^5^ ^,^ ^6^ ^,^ ^8^. This population group presents socioeconomic vulnerabilities, notably precarious housing and basic sanitation conditions, which are factors associated with the disease due to constant exposure to the mosquito vector in such areas where transmission chains are developed and maintained^19^, as was observed during fieldwork.

The expressive number of cases in rural areas may be related to a higher percentage of households and inhabitants, including those below the poverty line^20^. This situation may be associated with the recent disorderly occupation of rural areas in the municipality, which led to the creation of areas with high susceptibility to occurrence of malaria cases.

The thick drop/smear test was the most utilized technique in this study because of its sensitivity, low cost, capacity to detect parasitemia even at low values (between 40 and 60 parasites per 100 fields), ease of use, and its ability to identify the *Plasmodium* species. This is the most widely used test in the Amazon as it has high endemicity for malaria and difficulty with access to health service facilities due to long distances^25^ ^,^ ^26^. The observation that *Plasmodium vivax* was the most prevalent species in São Félix do Xingu may be related to two factors. First, the specific reproductive cycle characteristics of this etiological agent, considering the vector-host relationship in comparison to other species. Second, the ecology of the disease vectors, which show great environmental adaptation, including in areas affected by deforestation. This situation may have resulted in an increase in population density and transmissibility of this pathogen in the municipality, as shown in other studies in the Amazon region^11^ ^,^ ^19^ ^,^ ^27^ ^,^ ^28^. The laboratory identification of medium-risk malaria transmission in the Taboca and Ladeira Vermelha administrative districts may be related to the high density of cases in the localities of São Raimundo, Pista da Liberdade, Solar das Águas, and São Francisco. 

This fact suggests a possible vectorization of the disease with active transmission circuits, demonstrated by the kernel technique, due to anthropic processes such as the high deforestation observed in these areas. The evidence of this association may thus be due to the disorderly establishment of human settlements built for the implementation of agroextractivist activities, such as placer mining and pasture. During the fieldwork, a low socioeconomic level of the resident populations in areas with very high density of cases was also observed, in addition to the presence of natural and artificial breeding sites for malaria vectors^16^.

The conjunction of all these adverse socioeconomic and environmental factors, observed mainly in the localities of Pista da Liberdade and São Raimundo, is of major importance to public health because of their location near the boundaries of IL and CU that are under major human pressure from continuous deforestation processes^29^. This suggests the potential production of risk factors for the occurrence of several infectious diseases among the vulnerable populations of the municipality, most significantly malaria^30^.

The observation of the largest number of cases in areas with secondary vegetation, marked by the ecological succession of young and adult secondary forests known as *capoeiras,* which is mainly connected to the establishment of pastures. This was confirmed during fieldwork, where this type of land use was identified as the main driver behind the suppression of natural vegetation in the municipality, especially along the edges of PA and the resulting increase in pressure on them^29^. This situation highlights the need for implementing and expanding epidemiological and environmental surveillance in these areas because living there brings about an elevated level of susceptibility to the disease.

In this regard, the municipality of São Félix do Xingu has been ranked as one of the main cattle producers in Brazil since 2015, and its production area currently accounts for approximately 7% of the total deforestation in the state of Pará^9^. This situation reflects the issues and problems inherent to the advancement of such socioeconomic activities into the Amazon biomes and PA, which are factors associated with the environmental and socioeconomic production of malaria.

The evidence of a strong direct spatial autocorrelation between areas with high numbers of malaria cases and proximity to boundaries of full protection and sustainable use CU reflects the pressure placed on such areas in recent decades because of the effects of activities such as placer mining, which involves deforestation and degradation of water resources through the use of chemical products in mining processes, with the resulting establishment of breeding sites for immature anopheline forms that become vectors for malaria^5^ ^,^ ^6^ ^,^ ^31^ ^-^ ^33^.

The evidence of a weak inverse spatial autocorrelation between areas with ILs that present a low number of cases and the percentage of human impact next to these PAs, especially along their edges, may be related to the constitutional guarantee of possession that indigenous peoples have over their territories, given that their culture promotes valuing and preserving of the natural environment where they live. However, this may also indicate a hidden prevalence of malaria in these PA because of their inadequate and insufficient public health services, which leaves their populations even more vulnerable^4^ ^,^ ^34^.

In this study, the decreased number of malaria cases in the municipality may be related to the growing insufficiency of diagnostic tests in the Amazon in recent years^26^, implying that disease underreporting influences the generation of some information on epidemiological scenarios, such as the API. This fact has been aggravated by the precariousness of the populational basis at the local scale, including the administrative districts, constituting a limitation for carrying out studies on the environmental and socioeconomic production of parasitosis in the region. Thus, this situation has become a major epidemiological challenge and must be extensively studied.

## CONCLUSION

Regarding the analysis of the results obtained, malaria is a major health problem in São Félix do Xingu. The epidemiological profile observed was in line with the current trend of this disease occurrence in the Amazon. Fieldwork in the study area made it possible to confirm laboratory evidence of environmental and socioeconomic production of the disease, aggravated by precarious public health services.

A non-homogeneous spatial distribution was also observed in the administrative districts, with clusters of cases in rural locations resulting from high levels of anthropic relations, such as deforestation for the establishment of placer mining and pastures, especially close to CU and IL. Such a scenario highlights the need to intensify epidemiological and environmental surveillance in the municipality, particularly in its PA.
